# Platelet Function in Patients with Diabetes Mellitus: From a Theoretical to a Practical Perspective

**DOI:** 10.1155/2011/742719

**Published:** 2011-08-21

**Authors:** Nicholaos Kakouros, Jeffrey J. Rade, Antonios Kourliouros, Jon R. Resar

**Affiliations:** ^1^Johns Hopkins University School of Medicine, and Johns Hopkins Hospital Division of Cardiovascular Disease, Baltimore, MD 21287-073, USA; ^2^Imperial College, London SW7 2AZ, UK

## Abstract

Patients with diabetes mellitus have an increased prevalence of vascular disease. Pathologic thrombosis associated with atherosclerotic plaque rupture is a major cause of morbidity and mortality. Platelets are intimately involved in the initiation and propagation of thrombosis. Evidence suggests that platelets from patients with type 2 diabetes have increased reactivity and baseline activation compared to healthy controls. We review the pathophysiology of platelet hyperreactivity in DM patients and its implications in clinical practice, with particular focus on acute coronary syndromes, percutaneous coronary intervention, and novel antiplatelet agents.

## 1. Introduction

Diabetes mellitus, that affects over 25 million people in the US and an estimated 285 million worldwide, is associated with a significant burden of cardiovascular disease [1, 2]. Patients with type 2 diabetes mellitus (DM2) have a 2- to 4-fold increased risk of premature cerebral, coronary, and peripheral vascular disease that together constitute the leading cause of death in these patients [3, 4]. Unlike the diabetes-specific microvasculopathy, neuropathy, nephropathy, and retinopathy, the macroangiopathic process in patients with diabetes represents an accelerated but pathophysiologically similar process to atherosclerosis in nondiabetic subjects. Thrombotic events of these vascular lesions, particularly in the cerebral and coronary vasculature, can be life threatening. 

Normal blood flow and thromboresistance is dependent on vasomotion, blood corpuscular elements, plasma components, and their interaction with the endothelial surface. Rupture of an atherosclerotic plaque exposes subendothelial material that promotes platelet activation and the local initiation of the coagulation cascade that can lead to thrombus formation at the site of endothelial disruption. Acute vascular events, such as myocardial infarction and stroke, are due to such atherothrombotic events rather than gradual progression of luminal stenosis caused by atheromatous plaque.

Patients with DM not only have a greater atheromatous plaque burden but also a thrombotic diathesis that is in part due to changes in the coagulation system with increased levels of plasma fibrinogen, increased intravascular thrombin generation, and reduced fibrinolytic potential [5, 6]. Equally importantly, however, platelets from patients with diabetes mellitus have dysregulated signaling pathways that lead to an increased tendency to activate and aggregate in response to a given stimulus (platelet hyperreactivity). Platelet activation contributes to the pathology by not only triggering thrombus formation but also causing microcapillary embolization and release of constrictive, oxidative, and mitogenic substances that accelerate progression of local vascular lesions.

Platelet hyperreactivity and increased baseline activation in patients with diabetes is multifactorial and associated with biochemical factors such as hyperglycemia and hyperlipidemia, insulin resistance, and an inflammatory and oxidant state. We aim to review the factors associated with increased platelet reactivity in patients with diabetes mellitus, with a predominant focus on DM type 2. We also discuss the clinical relevance of platelet hyperreactivity in diabetic patients with acute coronary instability and the possible choices of antiplatelet agents to suppress platelet activity in this population.

## 2. Biochemical Factors Affecting Platelet Function in Diabetes

Hyperglycemia is the diagnostic hallmark finding in diabetes mellitus and is associated with macrovascular disease even in the prediabetic stage. Hyperglycemia, particularly postprandial, plays a significant role in the DM-associated development of cardiovascular disease as well as the DM prothrombotic state [7, 8].

In healthy subjects, without DM, the induction of acute hyperglycemia can lead to increased platelet reactivity and platelet activation as evidenced by increased markers such as soluble P selectin and CD40-ligand [9–11]. Exposure of platelets to hyperosmolar solutions also causes increased reactivity, suggesting that hyperglycemia may have a direct osmotic effect [12]. Both chronic and acute hyperglycemia causes *in vivo *activation of protein kinase C (PKC), a transduction pathway mediator for many proaggregatory platelet agonists [13]. Platelets from patients with DM, unlike those from healthy individuals, also manifest short-term activation of the calcium-sensitive PKC*β* isoenzyme by acute hyperglycemia even* in vitro*, in the absence of additional stimuli, indicating an inherent diabetes-related dysregulation of this pathway. A study of patients with type 2 DM undergoing percutaneous coronary intervention (PCI) found that improvements in glycemic control were associated with reduced platelet reactivity [14]. The clinical correlate of these changes is that even mild elevations in preprocedural fasting glucose associate with increased risk of mortality following PCI and, conversely, optimal preprocedural glycemic control (HbA1c < 7%) in type 2 DM patients is associated with improved clinical outcome [15, 16]. 

Recurrent episodes of hyperglycemia lead to the nonenzymatic interaction between the carbonyl group of the reducing sugar and the primary amino group of a protein leading to a cascade of reactions, the final result of which is a heterogeneous group of compounds known as advanced glycation end products (AGEs) [17]. Some of these AGE cause externalization of platelet membrane phosphatidylserine that leads to surface clotting factor activation and so directly enhance the thrombogenic state [18]. Similarly, the platelets of patients with diabetes have increased glycation levels of surface membrane proteins which cause decreased membrane fluidity and increased platelet sensitivity to agonists. [19, 20]. 

**Figure 1:**
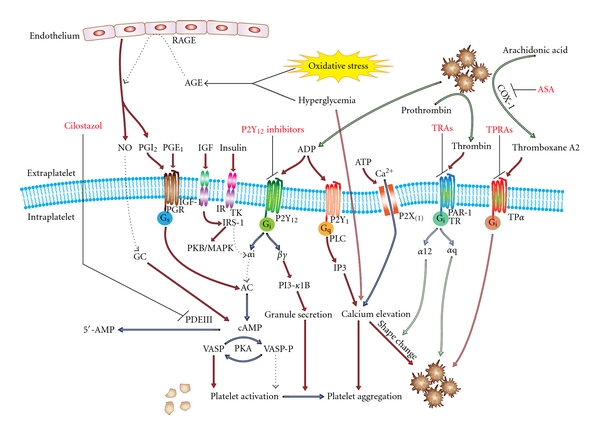
Pathways involved in platelet hyperreactivity in DM patients and therapeutic targets. AGE: advanced glycation end products, RAGE: AGE receptors, PKA/B/C: protein kinase A/B/C, MAPK: p38 mitogen-activated protein kinase, TK: tyrosine kinase, NO: nitric oxide, GC: guanylate cyclase, PAR-1 TR: protease activated receptor; thrombin receptor, PI-3: phosphoinositol-3 kinase TRA: thrombin receptor antagonist, TP*α*: thromboxane receptor, TPRA: thromboxane receptor antagonist, and ASA: acetylsalicylic acid (aspirin). Increased levels of cAMP lead to platelet inhibition through cAMP-dependent protein kinase (PKA) which inhibits signaling though the mitogen-activated protein kinases pathway, receptor activation, thromboxane A2 formation, and activation of key enzymes such as protein kinase C. The prostaglandin, P2Y, P2X, TR, and TP are all seven transmembrane G-protein associated receptors. The TR and TP on the right present novel drug targets; their intracellular effectors are omitted for clarity. Antiplatelet drugs are shown in red.

The final common pathway of platelet activation signaling is platelet aggregation mediated by the glycoprotein IIb/IIIa receptor (GPIIb/IIIa) platelet-fibrin interaction. The expression of platelet surface GP IIb/IIIa, as well as of GPIb, which mediates binding to von Willebrand factor, correlates with levels of hemoglobin A_1C_. Hyperglycemia leads to release of larger platelets with more GPIb and GPIIb/IIIa receptors and higher thromboxane forming capacity [21]. Other platelet surface receptors, such as P2Y_12_, the target of widely used thienopyridine antiplatelet agents, are also present in increased numbers on DM platelets likely as a result of the altered membrane fluidity dynamics [22]. Activation of the platelet P2Y_12_ receptor normally leads to reduced levels of cyclic adenosine monophosphate (cAMP) and subsequently suppressed phosphorylation of vasodilator-stimulated phosphoprotein (VASP-P) by specific protein kinases (PKA) that enhances platelet activation and aggregation (Figure 1) [23]. Platelets from patients with DM have lower levels of cAMP compared with nondiabetics, with consequently upregulated P2Y_12_ signaling. Platelets of older diabetics in particular have a higher baseline intracellular calcium level with more enhanced calcium mobilization from intracellular stores in response to thrombin agonism compared to nondiabetic patients [22, 24]. The higher baseline calcium and lower cAMP make the platelets more reactive such that they activate and aggregate at lower levels of agonist stimulation.

Abnormalities of lipid metabolism, particularly hypertriglyceridemia and low levels of high-density lipoprotein (HDL), are almost invariably found in patients with impaired glucose homeostasis. Hypertriglyceridemia can lead to triglyceride-rich very low density lipoprotein (VLDL) that potentiates platelet activity, an effect mediated partly through apolipoprotein E and an interaction with the platelet LDL receptor [25]. Interestingly, administration of reconstituted HDL to DM patients can promote cholesterol efflux from platelet membranes which suppresses aggregation [26]. Additionally, the interaction of lipids and glucose with the formation of glycated low-density lipoprotein (LDL) leads to impaired nitric oxide production and increased intraplatelet calcium concentration, further contributing to platelet hyperreactivity [27].

## 3. Insulin Effects on Platelets in Diabetes

Type 2 DM accounts for 90%–95% of all DM cases and is characterized by reduced tissue sensitivity to insulin. In the prediabetic stage this insulin resistance is initially met by a compensatory increase in insulin production by pancreatic *β*-cells sufficient to maintain fasting euglycemia. In susceptible individuals, the pancreatic *β*-cells, under the increased demand, undergo apoptosis leading to a reduction in *β*-cell mass. Consequently, the hyperinsulinemia characteristic of the early stages of DM2 progressively gives way to relative and eventually absolute insulin deficiency. 

Insulin can directly regulate platelet function via a functional insulin receptor (IR) found on human platelets [28]. The effects of hyperinsulinemia on platelets, however, are complex and disparate between normal individuals and patients with insulin resistance. *In vitro* experiments using platelets from healthy nonobese individuals reveal that binding of insulin to its receptor causes magnesium to translocate into the platelet and is associated with decreased thrombin-induced platelet aggregation and reduced production of proaggregatory thromboxane B2 [29]. Binding of insulin to the IR leads to activation of the insulin receptor substrate 1 (IRS-1) through tyrosine phosphorylation which initiates association with the G_i_ *α*-subunit. The result is reduced G_i_ activity that impairs tonic cAMP suppression, and thus leads to increased cAMP intraplatelet levels, blunting of P2Y12 signaling and reduced platelet activity (Figure 1) [30, 31]. It has been suggested that the magnitude of such effects may be limited due to the dimerization of IR subunits with those of the insulin-like growth factor 1 (IGF-1) receptor [32]. Nonetheless, the activation of insulin receptor substrate 1 (IRS-1) and IRS-2, the downstream mediator of the IR, can also occur by binding of IGF-1 to the IGF-1 receptor that is abundantly expressed in platelets leading to increased platelet reactivity [33]. Further, *in vivo*, experiments in healthy nonobese individuals confirm that insulin inhibits platelet interaction with collagen and attenuates the platelet aggregation effect of agonists [34, 35].

These findings suggest that at least in healthy nonobese individuals, insulin reduces platelet reactivity. Based on this, one may well expect increased platelet reactivity in DM type 1 patients who have absolute insulin deficiency. Similarly, one may postulate that patients with prediabetes or early stages of DM2 who have hyperinsulinemia should have suppressed platelet activity. This is, however, far from being the case. Obesity, a common feature in DM2 patients, can exacerbate or induce insulin resistance yet is associated with platelet hyperreactivity [36]. A euglycemic hyperinsulinemic clamp in obese insulin-resistant patients fails to suppress platelet activity even in the absence of overt DM [36]. Furthermore, obese patients have evidence of increased platelet activation with increased plasma CD40L, increased levels of platelet derived microparticles (released in blood by platelet activation), and higher thromboxane production [37–40]. Insulin sensitization by pioglitazone or weight loss reduces markers of platelet activation in obese women [37, 39]. 

The mechanism underlying these discrepant effects of insulin on the platelets of healthy individuals versus patients with insulin resistance appears to be impairment of the insulin receptor signaling pathway that occurs not only in tissues but also in platelets [22]. The reduced platelet insulin sensitivity leads to lower cAMP levels, increased intraplatelet calcium concentration, and platelet hyperreactivity [22, 41]. Indeed, insulin therapy in patients with type 2 DM may lead to paradoxical increases in platelet reactivity *in vivo* [42]. Additionally, platelets from DM patients show IRS-independent impairment of sensitivity to prostacyclin and nitric oxide that normally blunt platelet activation that leads to further increases in platelet reactivity [43, 44].

Hyperinsulinemia is, therefore, not protective but potentially detrimental to platelet reactivity in patients with insulin resistance. In addition to its platelet action, insulin has other adverse prothrombotic effects. Induced hyperinsulinemia, particularly in combination with hyperglycemia, leads to a procoagulant state by increasing levels of tissue factor procoagulant activity, decreasing factor VII/VIIa and increasing factor VIII and prothrombin fragment F1.2 [11]. In addition, there is upregulated platelet expression of CD40L and increased monocyte-platelet aggregates, indicative of platelet activation that appears distinct to the pathways of activation by other agonists [45].

## 4. Effects of Oxidative Stress and Inflammation on Platelet Function

Patients with DM have evidence of increased oxidative stress and inflammation compared with healthy subjects. DM is associated with an overproduction of reactive oxygen and nitrogen species and potent radicals, such as hydrogen peroxide and superoxide anion, that can directly lead to platelet activation [46–48]. DM patients have higher levels of 8-iso-prostaglandin F2*α* (8-iso-prostane), a product of nonenzymatic arachidonic acid peroxidation and marker of oxidative stress, particularly in association with acute hyperglycemic episodes [49–51]. Oxidative stress can directly affect platelet reactivity as superoxide anions enhance intraplatelet calcium release upon platelet activation, helping to amplify the platelet aggregation response [52].

The reactive oxygen species enhance the interaction of sugars with proteins during recurrent episodes of hyperglycemia and increase the rate of accumulation of previously mentioned advanced glycation end products (AGEs). These products can interact with AGE receptors (RAGEs) on the endothelium inducing endothelial dysfunction and an inflammatory response (Figure 1) [53]. Normal endothelium produces nitric oxide (NO) and prostacyclin which inhibit platelet activation under physiological conditions. Endothelial dysfunction leads to reduced production on NO and prostacyclin and so contributes to platelet hyperreactivity [54, 55]. NO can be oxidized by superoxide anions, leading to further reductions in its half-life and antiplatelet action [52]. 

Inflammation, associated with endothelial dysfunction, modulates the levels of proteins involved in platelet activation, such as increasing levels of the Fc*γ*-RIIA receptor that mediates enhanced activation in response to collagen [56]. Both oxidative stress and inflammation are also associated with accelerated turnover of platelets in patients with DM compared with healthy individuals, as indicated by the finding of immature, reticulated circulating platelets [57]. As platelet size correlates with activity, these large platelets are inherently hyperreactive and less responsive to antiplatelet therapy with aspirin and clopidogrel [58, 59].

## 5. Clinical Implications of Platelet Hyper-Reactivity in Diabetes

Low-dose aspirin remains the cornerstone of antiplatelet therapy by reducing the risk of MI, stroke, or cardiovascular death in intermediate-to-high-risk patients with established vascular disease by 20% [60]. Nonetheless, some patients' platelets remain reactive despite aspirin therapy when assessed by *in vitro* laboratory tests using a variety of platelet agonists. Multiple studies have found these patients to be at higher risk of atherothrombotic events [61–65]. The problem is particularly prominent in DM patients, 10%–40% of whom display high residual platelet reactivity on biochemical testing despite aspirin therapy [66, 67]. Although often termed “aspirin resistance”, this is, in most cases, a misnomer as failure of aspirin to achieve its expected pharmacological effect of inhibiting the conversion of arachidonic acid to TXA2 by the platelet cyclooxygenase-1 (COX-1) enzyme is actually quite rare with a prevalence of less than 5% in multiple studies [68, 69]. 

 A more appropriate and descriptive term for persistent agonist-induced platelet activation despite aspirin therapy is “high on-treatment platelet reactivity” (HTPR). Activated platelets produce the eicosanoid thromboxane A2 (TXA2), which creates a local positive-feedback loop that amplifies the activation response of the platelets to most agonists as well as activating bystander quiescent platelets. Aspirin irreversibly inhibits COX-1, the key enzyme in the conversion of arachidonic acid into TXA2 and thus interferes with the thromboxane feedback loop (Figure 1). Aspirin, therefore, limits the platelet response to weak agonists such as ADP and collagen though potent platelet agonists, such as thrombin, can still elicit strong platelet activation [70].

Due to the multiple pathophysiological mechanisms discussed above, patients with DM have hyperreactive platelets that are more sensitive to activation even by relatively weak agonists. As a result, even though aspirin may effectively block the TXA2 positive feedback loop, platelets of affected patients continue to manifest HTPR that places them at elevated risk of future thrombotic events. The inability of aspirin monotherapy to sufficiently blunt platelet reactivity provides the rationale for dual antiplatelet therapy in certain patients at high-risk of thrombotic events, such as patients with acute coronary syndrome (ACS) or undergoing PCI [71] and drives the search for more potent and specific antiplatelet agents with a more consistent platelet suppressing effect.

### 5.1. Thienopyridines—Clopidogrel

Similar to TXA2, ADP also exerts a positive feedback on platelet reactivity. Platelet activation leads to the release of adenosine diphosphate (ADP) from the dense granules that binds to platelet P2Y_1_ and P2Y_12_ receptors, potentiating platelet activation. The thienopyridines were the first class of alternative antiplatelet agents that became available and irreversibly inhibit the P2Y_12_ (but not the P2Y_1_) ADP platelet receptor, thus preventing the positive feedback amplification of agonist-induced platelet activation. The first thienopyridine compound, ticlopidine, was associated with noninsignificant rates of hematologic side effects, such as neutropenia and thrombotic thrombocytopenic purpura, and was superseded by the better tolerated second-generation agent, clopidogrel.

Patients with a high-risk of thrombotic complications, such as post-ACS or undergoing PCI, have been shown to benefit from aggressive dual antiplatelet therapy (DAT) consisting of aspirin in conjunction with clopidogrel. In the landmark *Clopidogrel in Unstable Angina to Prevent Recurrent Events (CURE)* study of ACS patients, patients with DM had a higher absolute risk reduction of primary outcome (16.7% to 14.2%) than non-DM patients (9.9% to 7.9%), albeit with a lower relative risk reduction from clopidogrel therapy compared to the overall cohort (15% versus 20%, resp.) [71, 72].

Clopidogrel does, however, have a number of drawbacks. It has a relatively slow onset of antiplatelet action and large inter-patient variability of platelet response due to variations in drug metabolism. Both ticlopidine and clopidogrel are prodrugs that require metabolic activation via a two-step process involving cytochrome P450 (CYP) isoenzymes. Polymorphisms in genes encoding for several of the CYP enzymes cause ineffective or even absent metabolic activation of clopidogrel in some patients resulting in HTPR. Patients with HTPR, despite treatment with aspirin and clopidogrel are known to be at increased risk of thromboembolic complications after drug eluting stent implantation [73–76]. Posttreatment platelet reactivity assessment with the VerifyNow P2Y_12_ system (Accumetrix, Inc., San Diego, Calif, USA) gives particularly good predictability of such thrombotic events [77]. 

DM has been found to be a predictor of HTPR despite clopidogrel therapy in a number of studies and populations [6, 78, 79]. In the *Optimizing Antiplatelet Therapy in Diabetes Mellitus *(*OPTIMUS*) trial, two thirds of diabetic patients were considered to have suboptimal response to aspirin and clopidogrel DAT, defined as a >50% residual platelet aggregation following ADP agonist stimulation. These poor responders had significant platelet reactivity suppression only with 150 mg daily doses of clopidogrel but not the usual 75 mg daily dose [80]. In a recent study, HTPR, despite clopidogrel therapy in patients with DM, was associated with a fourfold increase in periprocedural myocardial infarction compared to DM patients with suppressed platelet reactivity [81]. Interestingly, higher doses of clopidogrel (such as 600 mg loading dose) may overcome HTPR in some patients despite suboptimal glycemic control [82].

The HTPR problem may be further compounded by DM therapy. A recent study found that DM patients on sulfonylureas had a more than 2-fold higher rate of HTPR on clopidogrel, possibly due to competition of the two drugs for metabolism by the CYP2C9 cytochrome isoenzyme leading to reduced biotransformation of clopidogrel to its active metabolite [83].

It is thought that the principal underlying pathophysiological abnormality responsible for the increased rates of clopidogrel HTPR in DM is that platelets from patients with DM have lower levels of cAMP compared with nondiabetics. Lower baseline cAMP leads to upregulated P2Y_12_ signaling and, consequently, a lower degree of platelet inhibition by P2Y_12_ antagonists. Increasing baseline platelet cAMP levels would, therefore, seem a reasonable approach in reversing this inherent resistance of DM platelets to P2Y_12_ inhibition.

### 5.2. Use of Cilostazol to Overcome Clopidogrel Platelet Resistance in Diabetes

Cilostazol is a phosphodiesterase-3 inhibitor that increases intraplatelet cAMP levels and could therefore be theorized to help overcome platelet resistance to P2Y_12_ inhibition [84]. As expected, in the *ACCEL-RESISTANCE *study, cilostazol reduced platelet activity more effectively than doubling the standard dose of clopidogrel to 150 mg in patients with high platelet activity following clopidogrel loading [85]. Increased inhibition of platelet P2Y_12_ signaling by the addition of 100 mg cilostazol to DAT in type 2 DM patients following PCI was also demonstrated in the randomized double-blind placebo controlled *OPTIMUS-2* study [86]. This increased platelet inhibition translated into a favorable clinical outcome in the *DECREASE *registry of almost 3,100 patients treated with aspirin/clopidogrel DAT or DAT plus cilostazol following PCI with a drug-eluting stent [87]. Triple antiplatelet therapy (TAT) with cilostazol significantly reduced 12-month risks of stent thrombosis and MI compared with DAT without increased risk of bleeding complications.

Interestingly, the recent *CILON-T* trial recruited 960 patients with ACS receiving DES and randomized them in an open-label fashion to DAT (aspirin and clopidogrel) or TAT (aspirin, clopidogrel, and cilostazol) [88]. Approximately one third of these patients had DM and half presented with ACS. The addition of cilostazol enhanced DAT platelet inhibition but there was no difference in composite adverse events at 6 months in the entire cohort or in the subgroup of DM patients. The authors hypothesized that the positive chronotropic effect of cilostazol may have had deleterious effects in the acute post-ACS setting that masked any favorable antiplatelet action. Interestingly, a genomics subgroup analysis of *CILON-T* revealed that patients with specific cytochrome P450 polymorphisms leading to impaired clopidogrel activation had higher rates of HTPR if they were randomized to DAT, but not if they received TAT [89]. This important finding suggests that HTPR may arise from the dysregulation of platelet activation mechanisms, as found in patients with DM, independently of the patient's ability to activate clopidogrel. It is also noteworthy that female patients (at lower risk of events), and elderly patients (at higher risk of bleeding complications) had more favorable outcomes with DAT rather than with triple therapy including cilostazol.

Although cilostazol may have some beneficial effect in DM patients by improving platelet response to DAT, there are concerns regarding its use in ACS. It is, furthermore, contraindicated in patients with congestive heart failure, further limiting its utility in this acute setting.

### 5.3. Newer Antiplatelet Agents in DM Patients Undergoing PCI

Diabetic patients with ACS have been shown to benefit from an early invasive strategy by PCI and derive a greater benefit from powerful platelet inhibition with glycoprotein IIb/IIIa receptor antagonists (GPRAs) than patients without DM [90]. In real-world clinical practice, however, patients with DM are offered invasive therapy and GPRAs even less frequently than nondiabetics [91]. The reason for this discrepancy is unclear but may be due to the higher incidence of moderate-to-severe renal impairment in DM patients [92]. Furthermore, the role of GPRAs in the management of these patients may need to be revised in view of the emergence of newer, faster acting and potent antiplatelet agents.

**Prasugrel**

Prasugrel is a third-generation thienopyridine antiplatelet agent that has received approval by the US Food and Drug Administration (FDA) and the European Medicines Agency (EMEA). Like clopidogrel and ticlopidine, prasugrel is a pro-drug that requires metabolic activation. Unlike these other agents, however, prasugrel is activated by a one-step hepatic reaction that is unaffected by CYP polymorphisms [93, 94]. This leads to more rapid and consistent antiplatelet effects compared to either ticlopidine or clopidogrel. The *Trial to Assess Improvement in Therapeutic Outcomes by Optimizing Platelet Inhibition With Prasugrel-Thrombolysis in Myocardial Infarction 38 *(*TRITON-TIMI 38*) enrolled 13,608 patients with intermediate-to-high-risk ACS or ST elevation MI undergoing PCI who were randomized to clopidogrel versus prasugrel. A subgroup analysis of patients with DM from this study showed that these patients benefited from a higher reduction in the primary end point (a composite of cardiovascular death, myocardial infarction, and stroke) than subjects without DM (hazard ratio—HR 0.72 versus 0.86, *P* < 0.001), particularly if on insulin therapy (HR 0.63, *P* = 0.009). The DM patients also had no significant increase in hemorrhagic complications unlike the non-DM subjects [95]. Consequently, the net clinical benefit from prasugrel was greater for DM patients than for those without DM. Based on these findings, prasugrel was officially endorsed by the UK National Institute for Health and Clinical Excellence (NICE) in October 2009 for limited use in combination with aspirin in high-risk ACS patients undergoing PCI (http://www.nice.org.uk/TA182). Along with patients undergoing primary PCI for ST elevation MI (STEMI) and patients with prior stent thrombosis whilst on clopidogrel therapy, patients with DM were singled out as a patient group most likely to benefit from this enhanced antiplatelet therapy. In the USA, the joint ACC/AHA/SCAI guidelines endorse the use of prasugrel for patients with STEMI undergoing PCI unless this is contraindicated due to a prior history of stroke or transient ischemic attack [96].

**Nonthienopyridine P2Y_12_ Receptor Antagonis**

Nonthienopyridine P2Y_12_ Receptor Antagonis including ticagrelor, cangrelor and elinogrel, that do not require metabolic activation are also being investigated. The phase 3 *CHAMPION* trials of the short-acting intravenous reversible P2Y_12_ inhibitor *cangrelor* were terminated due to poor interim results, while a phase 3 trial of *elinogrel*, unique in having both an oral and intravenous preparation, is in the planning stage [97, 98]. *Ticagrelor*, a member of a new cyclopentyl triazolo pyrimidine class of antiplatelet drugs has been more extensively investigated. It is an orally active adenosine triphosphate (ATP) derivative that reversibly inhibits the P2Y_12_ ADP receptor. It provides more consistent platelet inhibition than clopidogrel, as it does not require metabolic activation that may be affected by genetic polymorphisms, and its metabolite is also an active P2Y_12_ inhibitor [99]. It is also a more potent agent, leading to 30% more platelet inhibition than clopidogrel [100] and has a more rapid onset and offset of antiplatelet effect [101]. The phase 3 *PLATO *(*PLATelet inhibition and patient Outcomes*) trial randomized more than 18,500 patients with STEMI or NSTEMI between clopidogrel and ticagrelor [102]. Overall, ticagrelor was associated with a lower composite endpoint of cardiovascular death, MI, or stroke with no increase in major bleeding. A subgroup analysis of PLATO data showed that DM patients with ACS, representing approximately 25% of the cohort, also benefited from a greater primary endpoint reduction with ticagrelor than clopidogrel irrespective of glycemic control. Additionally, these patients enjoyed a reduction of coronary artery bypass related bleeding, the latter being likely due to the reversible nature of P2Y_12_ inhibition by ticagrelor [103]. Side effects of note were asymptomatic ventricular pauses and dyspnea that proved clinically limiting and led to drug discontinuation in 1% of patients. Nonetheless, underweight patients (below their respective gender median), patients not on lipid-lowering drugs at randomization, and North American patients had notably lower or no benefit from ticagrelor treatment. The latter finding, postulated to be related to the higher dose of aspirin used in North American patients, led the US Food and Drug Administration (FDA) to decline initial approval of the drug pending further review of the data, expected later this year.

Notably, in both the *TRITON-TIMI 38* and the *PLATO* trials, the newer agents were predominantly compared to “standard” clopidogrel dosing consisting of a 300 mg load followed by 75 mg daily maintenance. In the more recent *OASIS-7 (CURRENT)* trial of ACS patients undergoing PCI, a “high-dose” clopidogrel regimen (600 mg loading and 150 mg daily for the first week after PCI) reduced thrombotic endpoints compared to the “standard” dosing and marginally improved a composite primary outcome of cardiovascular death, myocardial infarction, or stroke at 30 days post-PCI. Although this result is an analysis of the PCI subgroup it is consistent with a meta-analysis of previous studies [104]. None of the *TRITON-TIMI 38* and fewer than 20% of patients in the *PLATO* trial received 600 mg clopidogrel; consequently, the relative benefit of prasugrel or ticagrelor compared to high-dose clopidogrel remains uncertain. Nonetheless, in the *GRAVITAS* study 5,400 patients undergoing, predominantly elective, PCI had platelet function testing using the Verify Now assay (Accumetrics, Inc., San Diego, Calif, USA). Those found to have HTPR despite DAT, a finding significantly more prominent in the DM subgroup, were randomized to standard DAT or high-dose clopidogrel loading and double-dose clopidogrel daily for 6 months. Although patients who were treated with higher doses of clopidogrel tended to have lower P2Y_12_ platelet reactivity there was no difference in the primary endpoint of cardiovascular death, myocardial infarction, and stent thrombosis [105]. A proposed mechanism for this failure is that high-dose clopidogrel is insufficient to overcome platelet hyperreactivity in these patients who may fare better with more potent therapy such as with prasugrel, ticagrelor, or other novel agents.

As many of the deranged intracellular pathways in DM contribute to P2Y_12_ inhibition, it is hoped that agents that can inhibit alternative activation pathways may be helpful in DM patients. One such potential novel target is the protease-activated receptor 1 that mediates the effect of thrombin, the most potent physiologic platelet activator. Thrombin receptor antagonism has the additional theoretical benefit of blocking the cellular effects of thrombin without inhibiting the thrombin-mediated cleavage of fibrinogen (that is the final stent of the coagulation cascade) and may, therefore, cause less bleeding than other antithrombotic agents [106–109]. At least one such agent is currently being trialed in DM patients (clinicaltrials.gov: NCT00855374). Similarly, the thromboxane receptor provides a possible therapeutic target in blocking the TXA2 feedback loop. A TP selective antagonist is currently in phase III clinical trial for prevention of recurrent ischemic complications in patients with prior transient ischemic cerebral events [110].

### 5.4. Antiplatelet Therapy in DM Patients with Stable Vasculopathy

It must, finally, be noted that although DM patients have higher baseline platelet reactivity, this does not appear sufficient to warrant aggressive antiplatelet therapy in the chronically stable DM patient. DM patients with asymptomatic, stable coronary, cerebrovascular, or peripheral vascular disease did not have a reduction of MI, stroke or cardiovascular death with dual antiplatelet therapy (DAT) of aspirin and clopidogrel compared to aspirin alone in the *CHARISMA* trial [72]. DAT was, however, associated with increased hemorrhagic events. Interestingly, a posthoc analysis of this large trial suggested that, in the presence of diabetic nephropathy, the addition of clopidogrel to the antiplatelet regime of such patients was, in fact, associated with an increase in overall cardiovascular mortality, though the pathophysiological mechanism of this finding is unclear [111, 112]. Similarly, meta-analyses of the use of aspirin for the primary prevention of cardiovascular events suggest a modest reduction in cardiovascular events (MI and stroke) [113]. The data remain inconclusive to recommend aspirin use for primary prevention in all DM patients, though it should be prescribed to DM patients at >10% 10-year risk of cardiovascular disease and considered in patients at intermediate (5%–10%) risk according to the recent position statement of the American Diabetes Association [114] (Table 1).

## 6. Conclusion

Patients with DM have evidence of platelet hyperreactivity and increased baseline platelet activation. This results from a combination of factors including the effects of insulin, hyperglycemia, hyperlipidemia, endothelial dysfunction, oxidative stress, and inflammatory state. As our understanding of the molecular and genetic bases underpinning pathophysiological and therapeutic interactions expands, we can move closer towards individualized patient treatment.

Antiplatelet drugs interfere with platelet activation in the setting of pathologic atherothrombosis, but potentially also during physiologic hemostasis. There is currently no “magic bullet” antiplatelet agent that can effectively abolish atherothrombosis with no hemorrhagic penalty. The group of thrombin receptor antagonists is promising in this respect as this pathway may be of most significance in pathologic atherothrombosis rather than physiologic hemostasis. Currently bleeding is, therefore, a major factor to determine the risk-to-benefit utility of present and upcoming antiplatelet drugs. In view of the enhanced platelet functionality in DM patients, this ratio and, consequently, the choice of antiplatelet agents may differ from the general population. 

Patients with DM represent an important subgroup who may enjoy a greater net clinical benefit from a more potent antiplatelet regimen. The benefit DM patients derive from early invasive therapy and use of GPIIb/IIIa receptor antagonists is well established, but these agents are underutilized in clinical practice. The addition of cilostazol to aspirin/clopidogrel dual antiplatelet therapy in patients with DM merits consideration though there remain concerns for its use in the ACS DM population. The results from the large clinical studies of prasugrel (*TRITON-TIMI 38*) and ticagrelor (*PLATO*) appear favorable for the use of these agents in conjunction with aspirin following PCI in DM patients. Although analysis of the diabetic patient subgroup in these landmark studies was prespecified, randomization was not performed. Consequently, outcome data from trials of these novel antiplatelet therapies, specifically recruiting high-risk DM patients, are eagerly awaited.

##  Conflict of Interests

The authors declare that there is no conflict of interests.

## Figures and Tables

**Table 1:** A summary of key antiplatelet drug clinical trials in patients with diabetes mellitus.

Study	*N* (DM/total)	Setting	Groups	Endpoint. Follow-up period	Pertinent findings
Clopidogrel					

*CURE * Clopidogrel in unstable Angina to prevent recurrent events	2,838/12,562	UA, NSTEMI	Clopidogrel (300 mg LD, 75 mg MD) versus placebo in addition to standard aspirin therapy. RCT	Composite cardiovascular death, MI, or stroke. Mean followup 9 months	Higher absolute risk reduction of primary outcome (16.7% to 14.2%) in DM than non-DM patients (9.9% to 7.9%), but a clopidogrel benefit of borderline statistical significance with lower relative risk reduction compared to the overall cohort (15% versus 20% resp.)

*OPTIMUS *Optimizing antiplatelet therapy in Diabetes Mellitus	40/40	DM patients on DAT with HTPR	Clopidogrel 75 mg MD versus Clopidogrel 150 mg MD. RCT	Platelet function testing at 60 days	Two thirds (40/64) of screened DM patients had “suboptimal response to clopidogrel” 75 mg (HTPR). Platelet aggregation in response to ADP was significantly reduced in DM patients receiving clopidogrel 150 mg compared with the 75 mg group (*P* = 0.002)

*OASIS-7 (CURRENT) *Clopidogrel and Aspirin optimal dose usage to reduce recurrent events—seventh organization to assess strategies in ischemic syndromes	5,880/25,087	ACS patients planned for invasive strategy	Clopidogrel (600 mg LD, 150 mg MD for 6 days, then 75 mg MD) versus Clopidogrel (300 mg LD, 75 mg MD). Aspirin (300–325 mg MD) versus aspirin (75–100 mg MD). 2 × 2 factorial design	Composite cardiovascular death, MI, or stroke. 30 days	No overall statistical significance between the two clopidogrel regimes on primary endpoint. No significant difference between higher-dose and lower-dose aspirin with respect to the primary outcome. Reduced secondary outcome of stent thrombosis with high-dose Clopidogrel in subset of patients undergoing PCI

*GRAVITAS * Gauging responsiveness with a verifynow assay—impact on Thrombosis and safety	1,004/2,214	Patients post PCI with HTPR on DAT by verifynow assay	Clopidogrel (75 mg MD) versus Clopidogrel (repeat 600 mg LD, 150 mg MD)	Composite cardiovascular death, MI, stent thrombosis. Bleeding safety endpoint. 6 months	41% of screened patients (2214 of 5429) had HTPR on Clopidogrel 75 mg. Lower P2Y_12_ platelet reactivity with higher-dose Clopidogrel but no difference in the primary composite endpoint at 6 months

*CHARISMA *Clopidogrel for High Atherothrombotic risk and ischemic stabilization, management, and avoidance	6,556/15,603	Patients with stable cardiovascular disease or multiple risk factors	Clopidogrel 75 mg versus placebo in addition to aspirin 75–162 mg daily	Composite cardiovascular death, MI, stroke. Median 28 months follow-up	No difference in primary endpoint between aspirin and DAT. Increased hemorrhagic events with DAT. Increased mortality in patients with DM nephropathy treated with DAT

Cilostazol					

*ACCEL/RESISTANCE *Adjunctive Cilostazol versus high maintenance dose Clopidogrel in patients with Clopidogrel resistance	14/60	Patients with HTPR after clopidogrel 300 mg loading	Clopidogrel 75 mg + cilostazol 100 mg bd versus Clopidogrel 150 mg. All patients on aspirin 200 mg/day. RCT	Platelet function testing at 30 days	Adjunctive cilostazol reduced the rate of HTPR and intensified platelet inhibition as compared with high-maintenance dose clopidogrel 150 mg/day

*OPTIMUS-2 *Optimizing antiplatelet therapy in diabetes mellitus 2	20/20	DM patients on DAT (aspirin 81 mg, clopidogrel 75 mg daily)	Adjunctive Cilostazol 100 mg bd versus placebo. 2-week cross-over double-blind RCT design	Platelet function	Enhanced P2Y_12_ platelet receptor signaling inhibition with cilostazol in adjunct to standard DAT. Significant side effects with cilostazol with high rate of drug withdrawal

*DECREASE Registry * drug-eluting stenting followed by Cilostazol treatment reduces adverse serious cardiac events	867/3,099	Patients after DES implantation	DAT (aspirin,and clopidogrel) versus TAT (aspirin, clopidogrel and cilostazol). Registry	Death, MI and stent thrombosis. 12 months	Cilostazol significantly reduced the 12-month risk of stent thrombosis and MI after DES implantation when added to DAT. No increase in major or minor bleeding complications

*CILON-T * Influence of CILostazol-based triple antiplatelet therapy ON ischemic complication after drug-eluting stent implantation	307/960	Patients after DES implantation	DAT (aspirin and clopidogrel) versus TAT (aspirin, clopidogrel and cilostazol). Open-label, blind evaluation	Cardiac death, MI, ischemic stroke, and TLR at 6 months	Enhanced platelet inhibition with TAT but no difference in composite adverse events at 6 months (entire cohort or DM patients). Higher adverse events with TAT versus DAT in females and elderly patients

Prasugrel					

*TRITON-TIMI 38 *Trial to assess improvement in therapeutic outcomes by optimizing platelet inhibition With Prasugrel-Thrombolysis in Myocardial infarction 38	3,146/3,608	Patients with moderate-to-high-risk UA/NSTEMI, STEMI for PCI	Clopidogrel (300 mg LD and 75 mg MD) versus Prasugrel (60 mg LD and 10 mg MD)	CV death, MI and stroke. Bleeding safety endpoint	DM patients on prasugrel had higher reduction in endpoint compared to clopidogrel than non-DM patients (HR 0.72 versus 0.86, *P* < 0.001). Benefit of prasugrel was greater amongst DM patients on insulin (HR, 0.63; *P* = 0.009). Patients without DM had significantly increased risk of TIMI major hemorrhage on prasugrel versus clopidogrel (1.6% versus 2.4%; *P* = 0.02) but DM patients had similar bleeding rates on the two drugs (*P* = 0.81). Greater net treatment benefit with prasugrel versus clopidogrel in DM patients

Ticagrelor					

*PLATO * platelet inhibition and patient outcomes	4,662/18,624	Patients with moderate-to-high-risk UA/NSTEMI, STEMI for PCI	Clopidogrel (300 mg LD and 75 mg MD) versus Ticagrelor (180 mg LD and 90 mg bd MD)	CV death, MI and stroke. Bleeding safety endpoint. 6–12months	Ticagrelor was associated with a lower composite endpoint with no increase in bleeding in the entire cohort as well as DM patients. Effects were irrespective of DM status, insulin treatment, and glycemic control

ACS: acute coronary syndrome, ADP: adenosine diphosphate, bd: twice daily, DAT: dual antiplatelet therapy, DES: drug-eluting stent, DM: diabetes mellitus, HR: hazard ratio, HTPR: high on-treatment platelet reactivity, LD: loading dose, MD: maintenance dose, MI: myocardial infarction, NSTEMI: non-ST elevation MI, RCT: randomized control trial, STEMI: ST-elevation MI, TAT: triple antiplatelet therapy, UA: unstable angina.
